# ATM variants 7271T>G and IVS10-6T>G among women with unilateral and bilateral breast cancer

**DOI:** 10.1038/sj.bjc.6601289

**Published:** 2003-10-14

**Authors:** J L Bernstein, L Bernstein, W D Thompson, C F Lynch, K E Malone, S L Teitelbaum, J H Olsen, H Anton-Culver, J D Boice, B S Rosenstein, A-L Børresen-Dale, R A Gatti, P Concannon, R W Haile, the WECARE Study Collaborative Group

**Affiliations:** 1 Department of Community and Preventive Medicine, Mount Sinai School of Medicine, One Gustave L Levy Place, Box 1043, New York, NY 10029-6574, USA; 2 University of Southern California, 1441 Eastlake Avenue, Los Angeles, CA 90033, USA; 3 University of Southern Maine, PO Box 9300, Portland, ME 04104-9300, USA; 4 Department of Epidemiology, University of Iowa, C21-L GH, Iowa City, IA 52242, USA; 5 Fred Hutchinson Cancer Research Center, 1100 Fairview Avenue N., MP-381, PO Box 19024, Seattle, WA 98109, USA; 6 Danish Cancer Society, Strandboulevarden 49, Copenhagen DK-2100, Denmark; 7 University of California, Irvine, 224 Irvine Hall, Irvine, CA 92697, USA; 8 International Epidemiology Institute, 1455 Research Boulevard, Suite 550, Rockville, MD 20850-6115, USA; 9 Vanderbilt University, 2201 West End Avenue, Nashville, TN 37235, USA; 10 Norwegian Radium Hospital, Montebello, Oslo N-0310, Norway; 11 University of California, Los Angeles, 10833 LeConte Avenue, Los Angeles, CA 90095-1732, USA; 12 Virginia Mason Research Center, 1201 Ninth Avenue, Seattle, WA 98101-2795, USA

**Keywords:** ATM gene screening, 7271T>G mutation, IVS10-6T>G mutation, breast cancer, bilateral breast cancer

## Abstract

Recent reports suggest that two ATM gene mutations, 7271T>G and IVS10-6T>G, are associated with a high risk of breast cancer among multiple-case families. To assess the importance of these two mutations in another ‘high-risk’ group, young women (under age 51) with multiple primaries, we screened a large population-based series of young women with bilateral breast cancer and compared the frequency of these mutations among similar women diagnosed with unilateral breast cancer. The 1149 women included were enrolled in an ongoing population-based case–control study of the genetic factors that contribute to bilateral breast cancer; they were not selected on the basis of family history of cancer. Screening for 7271T>G and IVS10-6T>G ATM gene mutations was conducted using DHPLC followed by direct sequencing. The 7271T>G mutation was detected in one out of 638 (0.2%) women with unilateral breast cancer and in none of the bilateral cases, and the IVS10-6T>G mutation in one out of 511 (0.2%) bilateral and in eight out of 638 (1.3%) unilateral breast cancer cases. Carriers of either mutation were not limited to women with a family history. Given the likelihood that young women with bilateral breast cancer have a genetic predisposition, the observed mutation distribution is contrary to that expected if these two mutations were to play an important role in breast carcinogenesis among individuals at high risk.

ATM (for ataxia-telangiectasia (A-T) mutated), a gene whose product plays a critical role in signalling and responding to the presence of DNA double-strand breaks, is mutated in the autosomal recessive disorder A-T. The incidence of breast cancer among heterozygous carriers in A-T families, along with the known biochemical interactions between the products of the ATM and BRCA1 genes, has suggested a role for ATM in breast cancer risk. The recent study by Chenevix-Trench *et al* (2002) reported a greatly elevated risk of breast cancer among five members from multiple-case breast cancer families, who were heterozygous for ATM gene mutations, 7271T>G or IVS10-6T>G. The estimated combined penetrance of the two mutations was 60% (32–90%) to age 70 years. This is equivalent to a relative risk (RR) of 15.7 (95% confidence interval (CI)=6.4–38.0) compared to the general population. These findings are consistent with two earlier studies: one study of the British families that first identified the 7271T>G mutation and reported a similarly large increased risk of breast cancer among three carriers of that mutation (RR=12.7, 95% CI 3.7–45.8) (Stankovic *et al*, 1998); and a second study by Broeks (Broeks *et al*, 2000) of early-onset female breast cancer, where three out of the seven ATM mutations found were IVS10-6T>G. While not all studies of ATM gene mutations demonstrate an excess risk of breast cancer (FitzGerald *et al*, 1997; Bebb *et al*, 1999; Shafman *et al*, 2000), studies that have screened for missense mutations (Athma *et al*, 1996; Teraoka *et al*, 2001) and those that have examined risk among family members of A-T patients (obligate heterozygotes) (Swift *et al*, 1987, 1991; Pippard *et al*, 1988; Borresen *et al*, 1990; Inskip *et al*, 1999; Olsen *et al*, 2001) have consistently found an elevated risk. Combined, these results provide evidence for an increased breast cancer risk associated with specific ATM gene mutations.

Women with second primary breast cancer have an increased genetic susceptibility to breast cancer compared to women with unilateral breast cancer, or to the general population. Thus, any genetic abnormality that is important in the etiology of breast cancer will be considerably more prevalent among women who have had a first breast primary than in the general population and, in turn, even more common among women with bilateral breast cancer (Thompson, 1986; Begg and Berwick, 1997). So, examining the frequency of a genetic mutation among women with bilateral breast cancer and comparing it with the frequency among similar women with unilateral breast cancer may provide important clues as to its role in breast carcinogenesis.

Given the extremely high risk of breast cancer associated with two ATM gene mutations, 7271T>G and IVS10-6T>G, observed by Chenevix-Trench *et al* (2002) among multiple-case families, the question arises concerning the role they play in other genetically susceptible high-risk groups, including women with multiple primary cancers. In this study, we investigated the prevalence of these two ATM gene mutations in a large population-based series of women with bilateral breast cancer compared with the frequency among women with unilateral breast cancer.

## MATERIALS AND METHODS

The 1149 women included in this analysis were available from the ongoing multicentre, population-based case–control study of second primary breast cancer and gene–environment interactions, the Women's Environmental, Cancer, and Radiation Epidemiology (WECARE) Study. The study population currently includes 511 women with asynchronous bilateral breast cancer, who serve as cases, and 638 women with unilateral breast cancer, who serve as controls. All participants are identified through five population-based tumour registries (Los Angeles County Cancer Surveillance Program, Cancer Surveillance System of the Fred Hutchinson Cancer Research Center, State Health Registry of Iowa, Cancer Surveillance Program of Orange County/San Diego-Imperial Organization for Cancer Control, and the Danish Cancer Registry). Selection is independent of a woman's family history of cancer. Women eligible for inclusion were under age 55 at diagnosis of the first primary breast cancer (invasive, with localised or regional, but not metastatic disease) and were first diagnosed between 1/1/1985 and 12/31/2000. All epidemiologic information is ascertained using a structured telephone-administered questionnaire, and blood samples are drawn by a study phlebotomist. The questionnaire contains items addressing known and suspected risk factors for breast cancer, including the family history of breast cancer and personal demographics. Each participant provided informed consent in accordance with the Institutional Review Board at each study site that approved the study.

The bilateral breast cancer cases were diagnosed with contralateral breast cancer (*in situ* or invasive, any stage) at least 1 year after their first primary diagnosis. The second primary can be an *in situ* carcinoma as women with a history of breast cancer are more likely to be closely monitored for a second primary cancer. All women were alive at the time of contact and had no history of other cancers (except nonmelanoma skin cancer). For this analysis, we selected all WECARE Study participants who had completed their interview and donated a blood sample as of 11/10/2002.

The ATM gene-mutation analyses were conducted using a staged approach of initial screening by denaturing high-performance liquid chromatography (DHPLC) followed by nucleotide sequencing of exons yielding variant DHPLC profiles. DHPLC conditions were optimised using positive controls with known mutation status. Two independent readers scored all DHPLC and sequencing output traces. For quality-control purposes, we randomly selected 11% of the 1149 samples (69 bilaterals and 55 unilateral) to be rescreened, and obtained 100% agreement between the duplicate samples. All laboratory screening was performed blinded to sample characteristics (Bernstein *et al*, 2003). In all, 10 samples with IVS10-6T>G were haplotyped with microsatellite markers S1819 (Rotman *et al*, 1994), NS22 (Udar *et al*, 1999), S2179 (Vanagaite *et al*, 1995) and S1818 (Rotman *et al*, 1994), using standardised alleles (Mitui *et al*, 2003).

## RESULTS

Table 1

**Table 1:** Characteristics of 1149 young women with unilateral and bilateral breast cancer included in this study

**Characteristic**	**Bilateral (*N*=511)**		**Unilateral (*N*=638)**	
*Age at diagnosis (mean and range)*				
First primary	46	(25, 54)	46	(25, 54)
Second primary	50	(28, 68)	NA	NA
				
Follow-up (years) after diagnosis^a^(mean and range)	10.8	(2.4, 17.1)	10.5	(3.6, 16.9)
				
*Race (N and %)*				
White	486	95.1%	603	94.5%
Black	10	2.0	13	2.0
Asian or Pacific Islander	2	0.4	8	1.3
Native American, Aleut, or Eskimo	3	0.6	3	0.5
Other	10	2.0	11	1.7
				
*Family history of breast cancer (N and %)*				
Any first-degree relative	161	31.5%	128	20.1%
Mother	108	21.1	86	13.5
⩾One sister	76	14.9	53	8.3
⩾One daughter	1	0.2	1	0.2
				
Any second-degree relative	148	29.0%	149	23.4%
Maternal grandmother	27	5.3	30	4.7
Paternal grandmother	25	4.9	21	3.3
⩾One maternal aunt	63	12.3	70	11.0
⩾One paternal aunt	54	10.6	56	8.8

a Time between first primary diagnosis and interview date.

summarises the characteristics of the 1149 breast cancer cases. The percent of women with a relative with breast cancer was greater among women with two primaries compared to women with only one (any first-degree relative: *χ*^2^=19.74 (*P*<0.0001); any second-degree relative: *χ*^2^=4.66 (*P*<0.03)). This observation is consistent with previous studies suggesting that women with bilateral breast cancer have a genetic predisposition towards breast cancer, and supports our hypothesis that this population may be enriched for breast cancer susceptibility genes. Among the 1149 women in this study, the 7271T>G mutation was found in one out of 638 (0.16%) woman with unilateral breast cancer (Table 2

**Table 2:** Detailed characteristics of the 10 women with breast cancer, who were found to be heterozygotes for one of the two ATM gene mutations 7271T>G or IVS10-6T>G

				**Family history of breast cancer (number of affected relatives)**								
				**First-degree relative**				**Second-degree relative**				
**ATM mutation**	**Laterality**	**Age at d*x*^a^**	**Follow-up after d*x*^b^ (years)**	**Any**	**Mother**	**Sister**	**Daughter**	**Any**	**Maternal grandmother**	**Paternal grandmother**	**Maternal aunt**	**Paternal aunt**
7271T>G	Unilateral	41–45	⩽10	0	0	0	0	0	0	0	0	0
IVS10-6T>G	Bilateral	⩽40	>10	1	1	0	0	1	0	0	0	1
	Unilateral	⩽40	>10	0	0	0	0	0	0	0	0	0
	Unilateral	41–45	⩽10	0	0	0	0	0	0	0	0	0
	Unilateral	41–45	>10	2	1	1	0	0	0	0	0	0
	Unilateral	46–50	⩽10	1	1	0	0	0	0	1	0	0
	Unilateral	46–50	⩽10	0	0	0	0	2	0	1	0	1
	Unilateral	46–50	⩽10	0	0	0	0	1	0	0	1	0
	Unilateral	46–50	>10	1	0	1	0	2	0	1	0	1
	Unilateral	46–50	>10	0	0	0	0	0	0	0	0	0

a Age at diagnosis. For a subject with bilateral breast cancer, this is the age at first diagnosis; the second diagnosis was between 46 and 50 years of age.

b Time between first primary diagnosis and interview date, range 5.1–16.7 years.

). The IVS10-6T>G mutation was found in one out of 511 (0.19%) bilateral and in eight out of 638 (1.25%) unilateral breast cancer. Compared to women with unilateral breast cancer, the chance of being a carrier of either mutation among the bilateral breast cancer cases was 0.137 (95% confidence interval: 0.003–0.996). Full screening and quality control of the other 64 coding exons of the ATM gene was complete for seven of the 10 women. No other mutations were detected in these samples, although two silent polymorphisms were observed. All women with a mutation were aged 50 or younger at diagnosis of their first primary, all had invasive primary breast cancer, and all were Caucasian. Their family histories were diverse and moderate; one woman reported two affected first-degree relatives with breast cancer, three women had one affected first-degree relative, and four subjects reported at least one affected second-degree relative. The samples with IVS10-6T>G were haplotyped with microsatelite markers. Although phase was not determined, the data were compatible with the interpretation that each sample shares a haplotype with the homozygous IVS10-6T>G A-T patient described by Broeks *et al* (2003).

## DISCUSSION

In the present study, we focused on the frequency of the 7271T>G and IVS10-6T>G ATM gene mutations, because prior studies of multiple-case families found these two mutations to be associated with excess breast cancer. The 1149 women in our study were population-based, and all diagnosed with a first breast cancer at a young age (under age 51). We observed a higher prevalence of IVS10-6T>G among unilateral breast cancer cases than bilateral breast cancers. This frequency distribution among bilateral and unilateral breast cancer cases is consistent with the study by Broeks *et al* (2003), which also focused on early-onset breast cancer; two out of 49 unilateral breast cancer cases and one out of 33 bilateral breast cancer cases were found to carry the IVS10-6T>G mutation. However, in our study, the prevalence of this mutation was more than 20-fold lower than that observed in the Broeks study. In contrast, the family study conducted by Chenevix-Trench *et al* (2002) found no IVS10-6T>G mutations in the 262 breast cancer patients who were unselected for family history; two were observed in the 76 multiple-case breast cancer families. Similar to our study's 7271T>G mutation prevalence of 0.08% (one out of 1149), Chenevix-Trench *et al* (2002) observed one mutation among 525 cases and 381 controls (0.11%); all women included in the study were under age 40. The prevalence of this mutation was greater among the high-risk families where one out of 76 (1.3%) carried the mutation. The 7271T>G mutation, originally detected in a Scottish family (Stankovic *et al*, 1998), to our knowledge, has only been observed in countries with a heavy British population origin. Thus, the low prevalence of this mutation in our study may reflect population heterogeneity. Among the bilateral breast cancer cases, 23% were diagnosed with an *in situ* second primary breast cancer; however, the single carrier of the IVS10-6T>G with bilateral breast cancer was diagnosed with an invasive second primary, 9.3 years after her first breast cancer was diagnosed. Further of note, from the haplotyping data, it appears that the IVS10-6T>G variant is carried on an ancestral haplotype that is common to patients with both A-T and breast cancer. This concurs with the findings of Broeks *et al* (2000), who also reported that this haplotype is observed in normal individuals carrying IVS10-6T>G and may confer incomplete penetrance. Ancient founder ATM mutations have been associated with other ancestral haplotypes as well (Campbell *et al*, 2003).

Given the likelihood that young women with bilateral breast cancer may be carriers of inherited susceptibility alleles at loci such as ATM, and that they are more genetically predisposed than women with unilateral breast cancer, in our series, we would have expected a greater frequency of germline mutations in the bilateral group. However, the observed mutation distribution, a higher prevalence in unilateral breast cancers than in bilateral breast cancers, is contrary to this and to what we would have expected if these two mutations were to play an important role in breast carcinogenesis among such individuals at high risk. Nevertheless, in the absence of information on the presence of any other ATM mutations associated with breast cancer in these patients, these results need to be interpreted cautiously.

## CONCLUSION

We examined the prevalence of two ATM gene mutations among women with unilateral and bilateral breast cancer; one out of 638 unilateral cases carried the 7271T>G mutation, while one out of 511 bilateral and eight out of 638 unilateral breast cancer cases harboured the IVS10-6T>G mutation. Neither mutation was associated with family history of breast cancer, age, or laterality.

## Appendix

*WECARE Study Collaborative Group:* Principal Investigator: Jonine L Bernstein, PhD (Mount Sinai School of Medicine, New York, NY); Co-Principal Investigators: W Douglas Thompson, PhD, Chair of the Epidemiology and Biostatistics Core (University of Southern Maine, Portland, ME); Robert W Haile, Dr PH (University of Southern California, Los Angeles, CA); Leslie Bernstein, PhD, Chair of the Data Collection Core (University of Southern California, Los Angeles, CA); Patrick Concannon, PhD, Chair of the Laboratory Core (Virginia Mason Research Center, Seattle, WA). Coordinating Center (Mount Sinai School of Medicine): Susan L Teitelbaum, PhD (Project Director); Gertrud S Berkowitz, PhD (Epidemiologist); Xiaolin Liang, MD, MS (Informatics Specialist); Monica Katyal, MPH (Project Coordinator); Stephanie Skoler, MPH (Project Coordinator); Brooke Levinson, MPH (Project Coordinator); National Cancer Institute: Daniela Seminara, PhD, MPH (Program Officer). Laboratories: Virginia Mason Research Center – Sharon Teraoka, PhD (Laboratory Director), Eric R Olson (Laboratory Manager); University of Southern California – Anh T Diep (Laboratory Director), Nianmin Zhou, MD (Laboratory Manager), Yong Liu, MD (Director of Blood Processing); Norwegian Radium Hospital, Oslo, Norway – Anne-Lise Børresen-Dale, PhD (Laboratory Director), Laila Jansen (Laboratory Manager); Mount Sinai School of Medicine – Barry S Rosenstein, PhD (Laboratory Director), David Atencio, PhD (Laboratory Manager); University of California at Los Angeles, Los Angeles, CA – Richard A Gatti, PhD (Consultant), Midori Mitui, PhD (Lab Manager). Data-Collection Centers: University of Southern California – Laura Donnelly (Project Manager), Maya Mahue-Giangreco, PhD (Project Manager); Danish Cancer Society, Copenhagen, Denmark – Jørgen H Olsen, MD, DMSc (Director), Lene Mellemkjćr, MSc, PhD (Project Manager); Fred Hutchinson Cancer Research Center, Seattle, WA – Kathleen E Malone, PhD (Director), Noemi Epstein (Project Manager); University of California at Irvine, Irvine, CA – Hoda Anton-Culver, PhD (Director), Joan Largent, MPH (Project Manager); University of Iowa, Iowa City, IA – Charles F Lynch, MD, PhD (Director), Jeanne DeWall (Project Manager). Radiation Core: University of Texas, MD Anderson Cancer Center, Houston, TX – Marilyn Stovall, PhD (Dosimetry Laboratory Director and Chair, Radiation Core); New York University, New York, NY – Roy E Shore, PhD, Dr PH (Epidemiologist); International Epidemiology Institute, Rockville, MD and Vanderbilt University, Nashville TN – John D Boice Jr, ScD (Consultant). Epidemiology and Biostatistics Core: University of Southern California – Duncan C Thomas, PhD, Bryan M Langholz, PhD.
